# A New Medical Society

**Published:** 1901-11

**Authors:** 


### Society Notes.

### A New Medical Society.

The San Angelo District Medical Society was organized September 28th. Dr. S. L. S. Smith was elected President; Dr. Boyd Cornick, First Vice-President; Dr. F. F. Tucker, Second Vice-President; Dr. R. L. Green, Secretary, and Dr. Bascom Lynn, Treasurer. The Society will meet in San Angelo, twice a month.

